# A Moderated Mediation Model of Self-Concept Clarity, Transformational Leadership, Perceived Work Meaningfulness, and Work Motivation

**DOI:** 10.3389/fpsyg.2019.01756

**Published:** 2019-08-06

**Authors:** Sunyoung Oh, Sang-Choong Roh

**Affiliations:** Department of Psychology, Institute of Applied Psychology, Sungkyunkwan University, Seoul, South Korea

**Keywords:** perceived work meaningfulness, personality, self-concept clarity, work motivation, transformational leadership

## Abstract

The purpose of this study was to investigate the role of self-concept clarity in predicting employees’ perceived work meaningfulness. We proposed a moderated mediation model in which perceived work meaningfulness could mediate the relationship between self-concept clarity and work motivation, and transformation leadership could moderate this mediating relationship. We tested our hypotheses using data collected from 488 employees in various companies. Results showed that individual differences in self-concept clarity were positively related to perceived work meaningfulness, and perceived work meaningfulness mediated the relationship between self-concept clarity and work motivation. Furthermore, the strength of indirect relationship between self-concept clarity and work motivation via perceptions of work meaningfulness was stronger when transformational leadership was low than when it was high. Implications of our findings along with limitations of this study and directions for future research are also discussed.

## Introduction

Perceived work meaningfulness (i.e., the degree to which an employee feels that work activities are generally meaningful and valuable) has been recognized by researchers as a critical psychological state that is vital to work outcomes (Hackman and Oldham, 1980; May et al., 2004; Humphrey et al., 2007). Previous studies have shown that perceived work meaningfulness exhibits positive relations with work engagement (May et al., 2004; Demirtas et al., 2017), job performance (Frieder et al., 2018), career commitment and job satisfaction (Duffy et al., 2011), psychological well-being (Arnold et al., 2007), and organizational commitment (Jiang and Johnson, 2018). In addition, evidence provided by meta-analytic review suggests that perceived work meaningfulness is the most important psychological state through which motivational job characteristics can affect work motivation (Humphrey et al., 2007). Research has also revealed that perceived work meaningfulness can decrease the need for monetary compensation when making job choices (Hu and Hirsh, 2017), while the lack of work meaningfulness can lead to turnover intention (Leunissen et al., 2018), exhaustion, cynicism, and stress (for review, see Rosso et al., 2010; Schnell et al., 2013). Given the importance of perceived work meaningfulness associated with employees’ motivation, well-being, and effective work behaviors, it is important to identify factors that make work meaningful.

Previous studies have suggested that perceptions of work meaningfulness are grounded in individual characteristics such as personality traits, personal needs, and beliefs (Rosso et al., 2010; Barrick et al., 2013; Allan et al., 2016; Lysova et al., 2018; Martela and Riekki, 2018). For example, Barrick et al. (2013) have suggested that personality traits (e.g., openness) can serve as sources of work meaningfulness through pursuit of higher-order goal striving (e.g., autonomy striving) that also interacts with task and job characteristics. In addition, research has shown that job enrichment and work role fit are important for a work to be perceived as meaningful (May et al., 2004). Adverse working conditions are known to be negatively associated with work meaningfulness (Arnoux-Nicolas et al., 2016). Leadership also has been suggested as an important social work context that can facilitate or constrain employees’ experience of work meaningfulness (Cai et al., 2018). Particularly, transformational leadership can effectively motivate followers to pursue a purpose that transcends short-term goals and focuses on followers’ higher-order intrinsic needs (Judge and Piccolo, 2004). Therefore, the degree to which employees perceive their supervisor as transformational is closely related to their perceived work meaningfulness (Frieder et al., 2018).

Although these studies have significantly enhanced our understanding of sources of perceived work meaningfulness, many studies on how personality factors can serve as sources of work meaningfulness remain theoretical (e.g., Rosso et al., 2010; Barrick et al., 2013; Lysova et al., 2018). Thus, researchers have continued to call for more empirical research on sources of meaningful work (Martela and Riekki, 2018). In particular, empirical investigations of the link between self-related beliefs and work meaningfulness are lacking. Rosso et al. (2010) have called for research on the role of various aspects or conceptions of the self in exploring sources of work meaningfulness. They have suggested that “individuals are the ultimate arbiters of the meaning of their own work, as shaped through the lens of their unique perceptions and experiences” (p. 115).

To better understand the influence of self on perceptions of work meaningfulness, the present study tested the possibility that individuals’ self-concept clarity (i.e., the degree to which individuals feel a clear and coherent sense of themselves, Campbell et al., 1996) might play an important role in their interpretation of their work as purposeful and significant. Although scholars have emphasized that how individuals view themselves can strongly influence their experience of work meaningfulness (Rosso et al., 2010), such suggestion remains theoretical. In this study, we argue that individuals’ self-concept clarity could serve as an antecedent to perceptions of work meaningfulness which in turn can affect work motivation. Furthermore, we enriched our research model by examining transformational leadership as an important work context that might change the strength of the relationship between self-concept clarity and work meaningfulness. As previously stated, transformational leadership is an important work context that makes employees perceive work to be meaningful (Grant, 2012; Frieder et al., 2018). Based on this, it is important to consider transformational leadership as a boundary condition of the relationship between self-concept clarity and work meaningfulness. Thus, the purpose of the present study was to test a moderated mediation model in which the strength of indirect effect of self-concept clarity on work motivation through perceived work meaningfulness was dependent on the level of transformational leadership. We believe that results of this study could broaden our understanding of individual differences and the role of self in the experience of work meaningfulness underlying work motivation mechanism.

### Theoretical Background and Hypotheses

Self-concept clarity refers to the extent to which people feel certain about whether they possess a clear and coherent sense of themselves (Campbell et al., 1996). It is important to note that self-concept clarity does not refer to self-knowledge that is associated with the valence of self-beliefs. Campbell et al. (1996) have argued that, although self-concept clarity and self-esteem have some similarities, self-concept clarity is a relatively stable trait that is distinct from self-esteem. Self-concept clarity emphasizes a structural aspect of the self-concept (i.e., the extent to which contents of one’s self-concept are “clearly defined, internally consistent and temporally stable”; Campbell et al., 1996, p. 141), whereas self-esteem reflects evaluative components of the self-concept. Thus, self-concept clarity reflects how well one knows oneself, whereas self-esteem reflects how positively one feels about oneself (Lee-Flynn et al., 2011).

Previous research has accumulated compelling evidence that self-concept clarity is closely related to psychological adjustment and well-being (Campbell et al., 1996; Bigler et al., 2001; De Cremer and Sedikides, 2005; Slotter et al., 2010). For example, Lee-Flynn et al. (2011) have found that higher levels of self-concept clarity can predict lower levels of depressive symptoms 2 years later after controlling for initial levels of depressive symptoms and self-esteem. This suggests that individuals who have a clear self-concept can effectively cope with negative appraisals by using their confidently defined clear self-beliefs to guide decisions in dealing with threatening and uncontrollable stressors. However, individuals with confused self-concept lack self-knowledge to guide how to behave. In addition, high self-concept clarity promotes effective self-regulation in managing ego-threatening social conflict situation (Bechtoldt et al., 2010), and is positively associated with relationship satisfaction and commitment (Lewandowski et al., 2010).

Extending the adaptive functioning of self-concept clarity to organizational settings, we argue that the clarity of self-concept may facilitate employees experiencing meaningfulness at work, which in turn can enhance their work motivation. Although appraisals of work meaningfulness rely on self and task as well as organizational characteristics, the self is the one that crafts a sense of meaningful work by unifying with other variables (Schnell et al., 2013). Rosso et al. (2010) have suggested that the extent to which people believe that their work contributes to the fulfillment of their core values and true self-identities is closely related with feeling authentic connection to oneself, and this sense of self-connectedness can lead to a greater sense of meaningfulness. Consistent with this idea, McGregor and Little (1998) have found that the extent to which participants assess personal projects as consistent with core aspects of self-identity or self-concept (e.g., values, goals, competencies) can predict the sense of meaningfulness. These findings suggest that individuals’ perceived work meaningfulness is closely associated with the sense of self-connectedness based on core elements of their self-concepts. Based on these findings, it is possible that if individuals can clearly perceive their self-concept, they may have clear sources to experience work meaningfulness. Accordingly, whether individuals have certain or uncertain feelings of self-concept might greatly contribute to the ways that they experience work meaningfulness.

Regarding this possibility, evidence provided by the work of Schlegel et al. (2009, 2011) on the meaning in life is notable, considering that meaning in life and meaning in work share common ground. That is, perceptions of meaningfulness are highly subjective appraisals that rely on more or less conscious perceptions of a coherent self-system (Schnell et al., 2013). Schlegel et al. (2009) have found that participants with faster responses to true self-concept words report a higher sense of meaning in life, indicating that accessibility of true self-concept traits is significantly related to perceptions that life is meaningful. Furthermore, Schlegel et al. (2011) have found that participants who feel ease to describe their true selves report higher perceived meaningfulness of life, compared to those who find it difficult to describe their true selves. These results emphasize the effect of having highly accessible true self-concepts on the formation of a sense of meaningfulness. Based on these findings, it seems likely that an individual with higher self-concept clarity may have easily accessible true self-concepts such as values and beliefs which enables him or her to experience the meaningfulness of work. However, an individual who is uncertain of his or her self-concepts may find it difficult to sense work meaningfulness. Thus, individual differences in clarity of self-concept may be closely related to perceptions of work meaningfulness.

The relationship of self-concept clarity with sense of control and perceived meaningfulness further support this notion. Baumgardner (1990) has suggested that self-certain individuals who think they know what they can do and what they cannot do are confident in effectively coping with different situations, feeling a sense of control over their lives. Wu and Yao (2007) have also suggested that individuals with higher self-concept clarity have a clear knowledge of their characteristics, which makes them capable of taking appropriate action when confronting various situations, thus having higher sense of control. Furthermore, individuals who believe that they have the ability to exercise control in their environments can experience meaningfulness because such belief satisfies their need for autonomy (Rosso et al., 2010). Taken together, it seems likely that higher self-concept clarity might be associated with higher sense of control and in turn related to perceived meaningfulness. Thus, we hypothesize that individuals with higher self-concept clarity would perceive more work meaningfulness than those with lower self-concept clarity. Given that perceived work meaningfulness is a direct determinant of work motivation (Humphrey et al., 2007), we also hypothesize that perceived work meaningfulness could mediate the relationship between self-concept clarity and work motivation. Thus, we have the following hypotheses.

Hypothesis 1: Individual differences in self-concept clarity have significant relationship with perceived work meaningfulness.

Hypothesis 2: Perceived work meaningfulness mediates the relationship between self-concept clarity and work motivation.

This study investigated the role of transformational leadership as a boundary condition that might moderate the relationship between self-concept clarity and work meaningfulness. Leadership research has accumulated evidence that leaders play a crucial role in their followers’ experiences of work meaningfulness (Shamir et al., 1993; Grant, 2012; Cai et al., 2018). In particular, transformational leadership is known to be effective in framing and reframing meanings that followers give to their work (Grant, 2012). Transformational leadership refers to leadership behaviors that motivate followers to pursue a higher-order purpose and vision beyond short-term self-interests through idealized influence (charisma), inspirational motivation, intellectual stimulation, and individualized consideration (Bass, 1985; Judge and Piccolo, 2004). Highly transformational leaders have inspirational impacts on followers by highlighting important visions and providing meanings for followers’ work. Furthermore, their idealized influences and individualized consideration can help followers personalize visions into their work, which can lead followers to feel more meanings of their work (Grant, 2012). Similarly, evidence suggests that transformational leadership has impacts on followers’ perceived meaningfulness at work. It is particularly effective in heightening perceived meaningfulness of employees who have personality traits that constrain their experience of meaningfulness, which can consequently result in reduced variance in perceived work meaningfulness among followers (Frieder et al., 2018).

Given these strong effects of transformational leadership on followers’ perceived work meaningfulness, we suggest that high levels of transformational leadership might serve as work context that facilitates perceptions of meaningfulness of individuals who are low in self-concept clarity, thus reducing the strength of the relationship between individual differences in self-concept clarity and perceived work meaningfulness. Consequently, high levels of transformational leadership would weaken the indirect relationship between self-concept clarity and work motivation through perceived work meaningfulness. However, under low levels of transformational leadership that lack facilitative effects on employees’ meaning construction, employees’ experience of work meaningfulness would highly rely on employees’ certainty of self-concept. As such, individual differences in self-concept clarity should be significantly related to employees’ perceptions of work meaningfulness. Therefore, perceptions of work meaningfulness can significantly mediate the relationship between self-concept clarity and work motivation. Thus, we have the following hypothesis:

Hypothesis 3: Transformational leadership moderates the indirect effect of self-concept clarity on work motivation through perceived work meaningfulness, such that the strength of the indirect effect is weaker when transformational leadership is higher.

## Materials and Methods

### Participants and Procedures

Data were obtained from 488 employees of 12 companies in various industries, including automobile, banking, and content delivery networks in Seoul, South Korea. Questionnaires were initially distributed on-site to 532 employees prior to a training class by a research associate who was blinded to the hypotheses of this study. Participation was voluntary. Although ethics approval was not required per our institution’s guidelines and national regulations at the time when all data for this study were collected, data collection was carried out in accordance with recommendations of the American Psychological Association’s Ethics Code. Specifically, all participants were assured that their responses were anonymous and would be kept strictly confidential. In the cover page of the questionnaire, it was clearly stated that “all your responses in this survey will be collected anonymously and be kept confidential, and only be used for research.” From this survey, we collected data from 511 employees after omitting questionnaires with incomplete or invalid responses. We checked for outliers before conducting statistical analyses. Twenty-three cases were identified as univariate outliers with SPSS (version 19) box plot procedure. Multivariate outliers were not detected using Mahalanobis and Cook’s D statistics. After outliers were eliminated, the final data set consisted of 488 employees (324 males and 164 females) with an average age of 36.50 years and work experience of 9.66 years. Regarding education, 1.6% had a high school degree, 79.7% held a bachelor’s degree, and 17.9% held a postgraduate degree.

### Measures

English language questionnaires were translated into Korean following guidelines of cross-cultural translation (Beaton et al., 2000). All scales were rated using a 7-point Likert scale (1 = *disagree very much*, 7 = *agree very much*).^1^ Cronbach’s alpha for each scale is displayed in Table 1.

**TABLE 1:** Descriptive statistics and inter-correlations among observed variables.

**Variables**	***M***	**SD**	**1**	**2**	**3**	**4**	**5**	**6**	**7**	**8**	**9**
1. Gender	1.34	0.47	–								
2. Age	36.50	8.28	−0.47^*^	–							
3. Career-length	9.66	7.54	−0.36^*^	0.84^*^	–						
4. Status	2.66	1.46	−0.42^*^	0.74^*^	0.81^*^	–					
5. Education	2.16	0.41	−0.09^*^	0.13^*^	0.06	0.13^*^	–				
6. SCC	3.88	0.95	–0.01	0.04	–0.01	–0.01	0.01	(0.81)			
7. T-L	4.69	1.02	–0.01	–0.02	–0.04	0.01	–0.08	–0.04	(0.94)		
8. PWM	4.74	0.95	0.05	0.08	0.08	0.06	0.06	0.09	0.44^*^	(0.89)	
9. Work motivation	4.98	0.86	–0.08	0.15^*^	0.15^*^	0.13^*^	0.06	0.11^*^	0.41^*^	0.70^*^	(0.89)

*N* = 488. Gender was coded female (1) or male (0). Cronbach’s alpha is in parentheses. SCC = self-concept clarity, T-L = transformational leadership, PWM = perceived work meaningfulness. ^*^p < 0.05.

#### Self-Concept Clarity

We assessed self-concept clarity using six items from Self-Concept Clarity Scale (Campbell et al., 1996). An example item is, “My beliefs about myself often conflict with one another.” (reversed). Higher scores reflect greater self-concept clarity.

#### Perceived Work Meaningfulness

Perceived work meaningfulness was measured with five items from Roh and Suh (2014) addressing meanings that individuals found in their work. Of these five items, four were originally from the scale of Meaning at Work (Ashmos and Duchon, 2000) (e.g., “The work I do is connected to what I think is important in life.”). The other item was from Kinjerski and Skrypnek (2006), “I experience a match between the requirements of my work and my values, beliefs, and behaviors.”

#### Transformational Leadership

We measured transformational leadership using 10 items from the Multifactor Leadership Questionnaire (Bass and Avolio, 1995), as have others (Jung and Avolio, 2000; Barling et al., 2002). We drew two items from each of five subscales of idealized influence-attributed, idealized influence-behavior, inspirational motivation, intellectual stimulation, and individualized consideration. An example item is, “My supervisor seeks differing perspectives when solving problems.”

#### Work Motivation

We measured work motivation using a five-item scale with three items from Guay et al. (2000) that addressed enjoyment. We also used two items from Van de Ven and Ferry (1980) that addressed effort. Example items are: “I think that my work is interesting and fun” and “I put a lot of effort into my work.”

#### Control Variables

Based on prior research (e.g., Riordan et al., 2003), we included age, gender, education level, status, and career-length of employees as control variables. Multiple regression analyses showed that gender and career length were the only significant predictors of work motivation and perceived work meaningfulness, respectively. Thus, gender and career-length were included in all models as additional predictors.

### Analytic Strategy

To simultaneously test pathways among the latent variables in the proposed model, we used structural equation modeling in Mplus Version 5.0 (Muthén and Muthén, 2010). Following the two-stage approach of Anderson and Gerbing (1988), the measurement model was first tested and then the hypothesized structural model was tested. Model fit was assessed with comparative fit index (CFI), Tucker-Lewis index (TLI), standardized root-mean-square residual (SRMR), and root-mean-square error of approximation (RMSEA). It has been suggested that CFI and TLI values > 0.95, SRMR values < 0.08, and RMSEZ < 0.06 indicate a satisfactory fit (Hu and Bentler, 1999).

We tested the hypothesized model in two steps (Klein and Moosbrugger, 2000). First, we assessed the fit of a mediation model without the hypothesized interaction. Second, we tested the moderated mediational model with latent interaction by comparing it with a nested model in which the interaction was constrained to zero. If moderated mediation is present, the model fit should be significantly better when the interaction term was added. Since conventional fit indices were unavailable for latent interaction estimations, a log-likelihood difference test (Δ-2*LL*) was used to assess the improvement in model fit (Muthén and Muthén, 2010). For the analysis of moderated mediation, we used MLR estimator in Mplus that could generate estimates with standard errors robust to non-normality of observed variables (Muthén and Muthén, 2010). To facilitate the interpretation of results, we standardized all model variables before running the analyses, and reported unstandardized coefficients (Kim and Ferree, 1981).

## Results

Table 1 shows descriptive statistics, reliabilities, and zero-order correlations among variables. We conducted a confirmatory factor analysis (CFA) to assess the four-factor structure and discriminant validity among the variables. Results of CFA showed that all observed items loaded significantly onto their respective factors and that the four-factor model had reasonable overall model fit [χ^2^ (293) = 1011.25, CFI = 0.91, TLI = 0.90, SRMR = 0.06, RMSEA = 0.07]. Modification indices (MIs) reported in Mplus called for error covariance between some items to be freely estimated. Based on inspection of MIs, we identified eleven pairs of items from the same measure (but not across measures) that could potentially have covaried error term. We then made one modification to the measurement model, specifying the error covariance of the eleven pairs by using “WITH” statement. This modification was deemed reasonable because each paired items were similarly worded to measure the same factor (e.g., for transformational leadership, “My supervisor seeks differing perspectives when solving problems.” and “My supervisor suggests new ways of looking at how to complete assignments.”). Including these eleven additional free parameters in the model, results of CFA showed a significant improvement in values of fit indices, resulting in a good model fit [χ^2^(282) = 560.40, CFI = 0.97, TLI = 0.96, SRMR = 0.06, RMSEA = 0.05]. In addition, compared to the three-factor model in which we merged perceived work meaningfulness with work motivation to form a single factor [χ^2^(285) = 721.42, CFI = 0.95, TLI = 0.94, SRMR = 0.06, RMSEA = 0.06], the four-factor model provided significantly better fit, Δχ^2^(3) = 161.02, *p* < 0.05. These results indicated that the four-factor model could fit the data adequately.

To test the hypothesized model, we first tested a baseline model (full meditation) with perceived work meaningfulness as a mediator of the effects of self-concept clarity and transformational leadership on work motivation. The results showed that this model has an acceptable level of fit, χ^2^(333) = 628.74, CFI = 0.96, TLI = 0.96, SRMR = 0.05, RMSEA = 0.04. When testing partial mediation, we included paths from self-concept clarity and transformational leadership to work motivation. These direct paths were not significant. Furthermore, adding these paths did not significantly improve model fit, Δχ^2^(2) = 1.16, *ns*. This suggests that perceived work meaningfulness fully mediated the relationships between self-concept clarity and work motivation. It also mediated the relationship between transformational leadership and work motivation.

**FIGURE 1:**
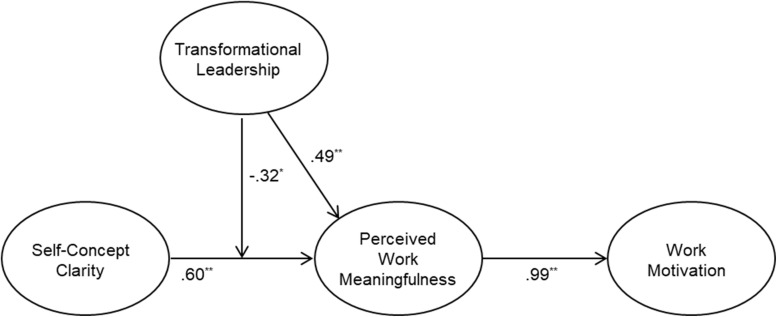
Moderated structural equation model results. Unstandardized coefficients are presented here. Paths of the control variables are omitted for clarity. ^*^p < 0.05, ^∗∗^*p* < 0.01.

We then tested a moderated mediational model with the latent interaction (Figure 1). The log-likelihood difference test demonstrated that adding the self-concept clarity × transformational leadership significantly improved the model fit, Δ-2*LL*(Δ*df*) = 5.16 (1), *p* < 0.05.^2^ Consistent with Hypothesis 1, the link between self-concept clarity and perceived work meaningfulness was significant. Other paths including the interaction term were also significant, as shown in Figure 1. We then tested the mediation predicted in Hypothesis 2, using procedure suggested by Preacher et al. (2007). Results revealed that the indirect effect of self-concept clarity on work motivation via perceived work meaningfulness was significant (indirect effect = 0.60, 95% CI = 0.29, 0.91), thus supporting Hypothesis 2.

**FIGURE 2:**
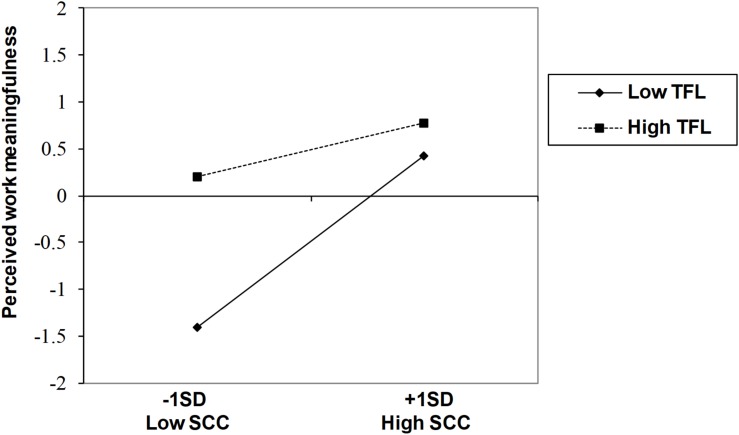
Interaction between self-concept clarity and transformational leadership on perceived work meaningfulness. SCC = self-concept clarity; TFL = transformational leadership. The value of zero on the vertical axis is the mean level of perceived work meaningfulness.

Following guidelines of Cohen et al. (2003), we plotted the interaction between self-concept clarity and transformational leadership. We also probed the interaction and examined the conditional indirect effect, using the procedure of Preacher et al. (2007). As shown in Figure 2, the relationship between self-concept clarity and perceived work meaningfulness was stronger when transformational leadership was low (*B* = 0.92, 95% CI = 0.37, 1.46) than when it was high (*B* = 0.28, 95% CI = 0.00, 0.56). Furthermore, consistent with the moderated mediation predicted in Hypothesis 3, the indirect effect of self-concept clarity was stronger when transformational leadership was low (conditional indirect effect = 0.91, 95% CI = 0.37, 1.45) than when it was high (conditional indirect effect = 0.28, 95% CI = 0.00, 0.56). Thus, Hypothesis 3 was supported.

## Discussion

### Overview of Findings and Theoretical Implication

This study tested a moderated mediation model to examine how self-concept clarity was related to work motivation through work meaningfulness in different levels of transformational leadership. Our results supported our proposed moderated mediation model. Specifically, we found that individual differences in self-concept clarity were positively related to perceived work meaningfulness, and perceived work meaningfulness mediated the relationship between self-concept clarity and work motivation. Furthermore, the indirect relationship between self-concept clarity and work motivation via work meaningfulness was stronger when transformational leadership was low than when transformational leadership was high.

These findings contribute to the self-concept clarity and work meaningfulness literature in several ways. First, our findings indicate that certain feeling of self-concept may contribute to fostering heightened perceptions of work as valuable and meaningful, suggesting that self-concept clarity is a valuable personality factor as an antecedent of employees’ perceptions of work meaningfulness in organizational settings. Although the self has been emphasized as a critical source of experience of work meaningfulness (Rosso et al., 2010), previous studies have not examined the role of self-concept clarity in exploring personality factors contributing to subjective meaning construction processes that underly motivated work behaviors. Our findings indicate that the degree to which individuals feel a clear and coherent sense of themselves plays an important role in their interpretation of their work as purposeful and significant. Thus, this study provides novel evidence to advance our understanding of personal characteristics that serve as sources of perceived work meaningfulness, which can impact work motivation.

In addition, it was notable that self-concept clarity was not significantly correlated with transformational leadership, although transformational leadership behaviors could enhance followers’ self-esteem and self-efficacy by engaging followers’ self-identity and self-worth (Kark et al., 2003; De Cremer et al., 2005; Walumbwa et al., 2008). Unlike self-esteem or self-efficacy that are associated with valence-related and evaluative aspects of the self (Campbell et al., 1996), it seems that self-concept clarity may not be affected by leadership behaviors that is known to be able to strengthen followers’ sense of self-worth. Based on the findings of the interaction between self-concept clarity and transformational leadership, we suggest that self-concept clarity may help explain why followers differ in responding to these leadership behaviors.

Second, this study expands the scope of research on individual differences in work motivation. Existing research has explored various personality characteristics that might be associated with work motivation, including personality traits and core self-evaluation (Judge and Ilies, 2002; Stanhope et al., 2013; Kanfer et al., 2017). However, few studies have investigated the effect of self-concept clarity to explain individual differences in work motivation. By demonstrating the importance of considering self-concept clarity in perceived work meaningfulness that drives work motivation, this study enhances our understanding of individual differences involved in work motivation.

Third, our findings make a contribution to transformational leadership literature by demonstrating transformational leadership as a boundary condition that can moderate the relationship between individual differences in self-concept clarity and perceptions of work meaningfulness. Recent research has revealed that transformational leadership has the moderating effects on the mediational relationship between outdoor salespeople’s personality traits (e.g., conscientiousness and openness) and their job performance through perceived work meaningfulness (Frieder et al., 2018). Consistently, the present study found that highly transformational leadership could effectively weaken the indirect relationship between individual differences in self-concept clarity and work motivation via work meaningfulness by heightening perceived work meaningfulness of employees who had less certain self-concept. Taken together, these results suggest that transformational leadership can serve as an external source to help employees perceive their work as being personally meaningful.

### Limitations and Future Directions

This study has several limitations. First, our data were collected using a cross-sectional, self-report survey which limited our ability to draw causal inferences. The research method also had the possibility of common-method bias. Future research could use experimental and longitudinal research designs to precisely test the claimed causality. In addition, to reduce common-method bias, future research could collect data from multiple sources. For example, future research may use coworker ratings of transformational leadership. While we used self-ratings of work motivation as an important work outcome, future research could include actual job performance and supervisor or coworker assessments of workplace adjustment.

Second, we did not control for personality traits that could affect perceptions of work meaningfulness and work outcomes. Given that recent findings have emphasized the role of personality traits as dispositional determinants of an individual’s experienced meaningfulness (Frieder et al., 2018), future research could include personality traits to investigate the unique contribution of self-concept clarity to perceived meaningfulness after controlling for personality traits. In addition, given that personality traits and self-concept clarity are associated with self-efficacy (Judge and Ilies, 2002; Wu et al., 2010), future research may need to explore whether and how self-concept clarity is related to personality traits in perceptions of self-efficacy that can affect the experience of work meaningfulness.

Third, we were unable to include task characteristics. Task identity (the extent to which an individual can complete a whole piece of work) and skill variety (the extent to which an individual must use different skills to perform his or her job) can affect the experienced meaningfulness associated with behavioral and attitudinal outcomes (Humphrey et al., 2007). These task variables may moderate the relationship between individual differences in self-concept clarity and perceived work meaningfulness. Thus, future research may include task characteristics to expand our understanding of work conditions interacting with self-concept clarity that can affect the experience of work meaningfulness.

## Ethics Statement

An ethics approval was not required for this study as per applicable institutional and national guidelines and regulations as research ethics committees (e.g., Institutional Review Board) were not available at the time our study was conducted. The informed consent of the participants was implied through survey completion. Data collection process was carried out in accordance with recommendations of the American Psychological Association’s Ethics Code. Specifically, all participants were assured that their participation was voluntary and that their anonymous responses would be kept strictly confidential. In the cover-page of the questionnaire, it was clearly stated that “all your responses in this survey will be collected anonymously and be kept confidentially, and only be used for research.”

## Author Contributions

All authors listed have made a substantial, direct and intellectual contribution to the work, and approved it for publication.

## Conflict of Interest Statement

The authors declare that the research was conducted in the absence of any commercial or financial relationships that could be construed as a potential conflict of interest.

## Footnotes

1 The Korean version of the scales and all data of this study are available from the first author upon request.

2 The output of this analysis is available from the first author upon request.
